# Potential of *Trichodermaharzianum* for control of banana leaf fungal pathogens when applied with a food source and an organic adjuvant

**DOI:** 10.1007/s13205-015-0327-0

**Published:** 2016-01-05

**Authors:** Suren Samuelian

**Affiliations:** Department of Agriculture, Fisheries and Forestry, Centre for Wet Tropics Agriculture, South Johnstone, QLD 4859 Australia

**Keywords:** Native *Trichoderma* spp., Antagonism, Biological control

## Abstract

*Trichoderma* isolates were obtained from diseased leaves and fruit collected from plantations in the main banana production area in Northern Queensland. Phylogenetic analyses identified the *Trichoderma* isolates as *T. harzianum* and *T. virens*. The *Trichoderma* spp. were found to be antagonistic against the banana leaf pathogens *Mycosphaerella musicola*, *Cordana musae*, and *Deightoniella torulosa* in vitro. Several products used by the banana industry to increase production, including molasses, Fishoil and Seasol, were tested as food source for the *Trichoderma* isolates. The optimal food substrate was found to be molasses at a concentration of 5 %, which when used in combination with a di-1-p-menthene spreader-sticker enhanced the survivability of *Trichoderma* populations under natural conditions. This formulation suppressed *D. torulosa* development under glasshouse conditions. Furthermore, high sensitivity was observed towards the protectant fungicide Mancozeb but Biopest oil^®^, a paraffinic oil, only marginally suppressed the growth of *Trichoderma* isolates in vitro. Thus, this protocol represents a potential to manage banana leaf pathogens as a part of an integrated disease approach.

## Introduction

Loss of sensitivity and development of fungicide resistance is becoming a worldwide problem across a range of fungicides and micro organisms (Eckert et al. 1994; Holmes and Eckert 1999; Karaoglanidis et al. 2001; Sholberg and Haag 1993). Currently control of leaf fungal pathogens on bananas (*Musa acuminata*) in Australia is achieved with the alternation of protectant fungicides, such as Mancozeb, chlorothalonil, paraffinic oils, and systemic fungicides belonging to the strobilurin (Q_o_I) and demethylation inhibiting fungicides (DMI), including the triazoles. The goal of such a strategy is to delay fungicide resistance. However, it has been shown that a shift in sensitivity or appearance of resistant strains occurs within 5 years of commercial introduction of a systemic fungicide (Marín et al. 2003). Such shifts have been documented for all Q_o_ inhibitors including trifloxystrobin, azoxystrobin, famoxadone, strobilurin B and myxothiazol (Chin et al. 2001; Sierotzki et al. 2000), and the triazoles propiconazole (Romero and Sutton 1996) and tebuconazole (Grice and Peterson 2002). Extensive use of benomyl (benzimidazole) by the banana industry worldwide resulted in its withdrawal from control programs (Romero and Sutton 1998; Stover 1977; Stover et al. 1978).

Demand for environmentally safe control measures has promoted the interest in finding non-chemical alternatives, such as biological agents, for the management of fungal pathogens. Most research on biological agents to control banana leaf diseases has been done with bacteria, but with unsatisfactory results under commercial conditions (reviewed by Marín et al. 2003), as this approach requires direct contact with the fungal phytopathogens. Jiménez et al. (1987) evaluated 225 epiphytic populations but with little success as these microorganisms experience difficulties in surviving on the phylloplane. A commercial strain of *Bacillus subtilis* (in Marín et al. 2003), *Serratia marcescens* isolates (Miranda 1996), and chitinolytic bacteria (González et al. 1996) evaluated under field conditions did not provide an effective control of banana leaf diseases.

Best results to improve biocontrol of a broad range of phytopathogenic fungi have been achieved with various strains of *Trichoderma* species (reviewed by Benítez et al. 2004). Schilly et al. (2014) explored 360 bacterial and 143 fungal banana root-associated endophytic microorganisms and found that *T. asperellum* significantly inhibited the growth of *Fusarium oxysporum* f. sp. *cubense*, the casual agent of Fusarium wilt of banana, a devastating problem for the banana industry worldwide. Genus *Trichoderma* possesses antagonistic properties, which are based on the activation of multiple mechanisms, acting synergistically. They affect fungal pathogens either directly by mycoparasitism, or indirectly by competing for nutrients and space, changing the microenvironment, inducing localised or systemic plant defences, acting like plant hormones, boosting plant rooting, and helping improve nutrient uptake (biofertilisation) resulting in increase plant growth (Benítez et al. 2004; Harman et al. 2004). Furthermore, *Trichoderma* spp. have been found to be highly resistant to a variety of chemical fungicides, toxins and xenobiotic compounds including antibiotics (Harman et al. 1996). Thus they might represent an opportunity to control banana leaf pathogens under commercial conditions.

Most studies have focused on biological agents as alternatives to synthetic fungicides and not as part of an integrated management system (Jacobsen et al. 2004). However, from the research done on biological control of leaf spot diseases (reviewed by Marín et al. 2003), it is evident that application of biological agents might not be sufficient for a successful disease management alone but instead biological agents have to be a part of an integrated management program (Elad et al. 1995; Harman 2000). The combination of biological agents with reduced levels of fungicides promotes a degree of disease suppression similar to that achieved with full fungicide treatment (Elad et al. 1993a, b; Monte 2001) and minimises development of fungicide resistance. Furthermore, locally occurring isolates will normally be more effective than isolates collected from different environment due to environmental adaptation. The aims of this study were (1) to identify *Trichoderma* strains that survive on leaves of *Musa acuminata* under wet tropical conditions, (2) to investigate their antagonistic potential against banana leaf fungal pathogens in vivo and in vitro, (3) to develop a strategy that will support the colonisation of banana leaves by *Trichoderma* populations under natural conditions, and (4) to explore the suitability of incorporating *Trichoderma* spp. as a biological agent in an integrated management program.

## Methods

### Fungal isolates and phylogenetic analysis

The main banana production area in Australia is the Tully-Innisfail coastal part of North Queensland (NQLD), which experiences high rainfalls (3334.6 mm per year) and tropical temperatures (mean maximum temperature 28.1 °C, and mean minimum temperature 19.2 °C (http://www.bom.gov.au/climate/). Fifty banana diseased leaf and fruit samples were collected from randomly chosen plantations from this region. Fungal isolations were carried out according to Pitt et al. (2010) with small modifications. Briefly, plant tissue with visible disease symptoms were surface sterilised with 70 % ethanol and left to air dry. Tissue sections were cut into pieces of approximately 5–10 mm^2^ and plated onto 90-mm standard Petri dishes (www.technoplas.com.au) containing Potato Dextrose Agar (PDA; Becton, Dickinson and Company, Difco^TM^) amended with 50 μg/mL streptomycin sulphate (Sigma-Aldrich, http://www.sigmaaldrich.com) after sterilisation. Samples were incubated for 40–48 h at 26 °C in the dark until actively growing mycelium was observed. Morphologically similar cultures were obtained by transferring hyphal tips from actively growing colonies onto fresh PDA plates. Sporulation for each isolate was induced by incubating fungal cultures at 25 ± 1 °C under diurnal light (12 h dark and 12 h near ultraviolet). Fungi were identified on morphological bases (conidial size, shape, and colour) and included *Mycosphaerella musicola* (yellow Sigatoka), *Cordana musae* (Cordana leaf spot), and *Deightoniella torulosa* (Deightoniella leaf spot) (Table 1). Fungal colonies with typical *Trichoderma* spp. characteristics such as green appearance, fast growth, and concentric green centres surrounded by white mycelium were selected and the genus confirmed microscopically according to Barnet and Hunter (1998). Molecular identification to species level of the *Trichoderma* isolates was conducted at the Plant Pathology Herbarium (BRIP), Queensland Department of Agriculture Fisheries and Forestry (QDAFF, http://collections.daff.qld.gov.au) based on the rDNA internal transcribed region (ITS; White et al. 1990). Two *Trichoderma* isolates were identified from a plantation in the Tully region of QLD, and one from the Centre for Wet Tropics Agriculture (CWTA), South Johnstone, QLD (near the town of Innisfail) (Table 1).

**Table 1:** Fungal isolates included in this study

BRIP^a^	Specie	Cultivar	Geographic origin	Date of isolation	GenBank Accession Nr.
60169^b^	*Trichoderma virens* (teleomorph: *Hypocrea virens*)^d^	Cavendish	Tully valley, QLD	2013	KM246885
60170^b^	*Trichoderma harzianum* (teleomorph: *Hypocrea harzianum*)^d^	Cavendish	Innisfail, QLD	2013	KM246886
60384^c^	*Trichoderma harzianum* (teleomorph: *Hypocrea harzianum*)^d^	Cavendish	Tully valley, QLD	2014	KM246887
60375^b^	*Cordana musae*	Cavendish	Tully valley, QLD	2013	
60374^b^	*Deightoniella torulosa*	Cavendish	Mount Bartle Frere, QLD	2013	
	*Mycosphaerella musicola*	Cavendish	South Innisfail, QLD	2014	

^a^Pure cultures of single spore isolates are deposited in the QDAFF Biological Collection (QDAFF, http://collections.daff.qld.gov.au)

^b^Isolates were obtained from leaves of banana, *Musa acuminata*

^c^Isolate was obtained from fruit of banana, *Musa acuminata*

^d^In this article the more common generic name *Trichoderma* is used over the younger name *Hypocrea*, the teleomorph of *Trichoderma* (Chaverri and Samuels 2001; Rossman et al. 2013)

Multiple sequence alignments and comparisons and contig assembly were performed with DNASTAR (www.dnastar.com). Nucleotide homology search and comparisons of identical sequences were performed with BLAST on publicly available databases at the National Center for Biotechnology Information (NCBI; www.ncbi.nlm.nih.gov). Genetic distances were calculated and maximum parsimony trees constructed with MEGA v. 5.0 (Tamura et al. 2007). Nucleotide sequences obtained in this study were deposited at NCBI (Table 1).

### In vitro assessment of antagonistic effect of *Trichoderma* agents against banana leaf pathogens

The *Trichoderma* isolates obtained in this study were analysed for their ability to suppress banana leaf fungal pathogens in vitro utilising the confrontation technique of Bell et al. (1982). Degrees of antagonism were scored on a scale of classes 1-5 representing different overgrowing capabilities of *Trichoderma* species as species belonging to classes 1 and 2 are considered to be highly antagonistic to the paired pathogen, class 3 are intermediate antagonists, and classes 4 and 5 are not antagonistic. All fungal cultures were grown on PDA except *M. musicola*, which was grown on V8 300-medium plates [300 mL V8 vegetable juice, 3 g CaCo_3_, 15 % Bacto Agar amended with 50 μg/mL streptomycin sulphate (Abadie et al. 2008)]. Comparisons were conducted by pairing 5-mm actively growing mycelium disks of a *Trichoderma* isolate and a plant pathogen on opposite sides of a Petri dish. Isolates of *C.* *musae* were started simultaneously with *Trichoderma* spp., *D. torulosa* was placed on agar 48 h before the *Trichoderma* spp., and *M. musicola* isolates were grown for 4 weeks until the colonies reached a diameter of approximately 18 mm before *Trichoderma* spp. were added to the plates.

### In vitro assessment of fungicide sensitivity

Mancozeb in conjunction with Biopest Oil^®^ at 2.5-5 L/ha (Table 2) are the banana industry standards for control of leaf diseases. An in vitro experiment was conducted to investigate the inhibitory effect of Mancozeb alone, Biopest Oil^®^ alone at 2.5 L/ha and 5 L/ha, and Mancozeb with Biopest Oil^®^ on the *Trichoderma* isolates identified in this study. The concentrations tested were in accordance to the dosage of application recommended by the manufacturers. Amended plates were prepared by adding sterifiltrated fungicide chemicals to melted and warm PDA at 50 °C before pouring the media into plates (Mondal et al. 2005; Samuelian et al. 2012). The centre of each plate was inoculated with a 5-mm agar plug of mycelium obtained from the edge of an actively growing PDA culture, with 6 replicates per treatment. Plates were maintained at 25 ± 1 °C under diurnal light to enhance sporulation. Simultaneous measurements were taken for all treatments and each individual fungal isolate when the growth of the no-fungicide control covered 80–90 % of the plates. The colony diameter was measured across two perpendicular axes. The response variable was the ratio of the average of the diameters for each treatment in comparison to the control (no-fungicide) treatment within the same isolate, set and replicate. This quantified the inhibition in fungal growth for each treatment in comparison to the control. Conidia were enumerated with a haemocytometer as previously described (Samuelian et al. 2014) as three counts were carried out for each replicate. Results were expressed as percentage growth compared to cultures grown on the no-fungicide control. The experiment was conducted twice.

**Table 2:** Formulation, rate of application and origin of the fungicides used in this study

Common name	Formulation^a^	Application rate (ai/ha)^b^	Product name	Supplier
Mancozeb	750 g/L, w.p.	1650 g	Penncozeb 750 DF	Nufarm
Paraffinic oil	l.	2.5–5 L	Biopest oil^®^	Sacoa
Fishoil	l.	8 L	Fishoil	Earth care
Seasol	l.	4 L	Seasol	Earth care
di-1-p-menthene	l.	600 mL	NuFilm-17	Agspec
di-1-p-menthene	l.	600 mL	FleXstic	Agritec

^a^Type of formulation: l.—liquid; w.p.—wettable powder

^b^Standard fungicide application dosage with ground equipment recommended by manufacturers is 250 L/ha

### In vitro assessment of best formulations of food substrate and other adjuvants

The Australian Banana Industry utilises stockfeed molasses at 5–20 %, Seasol at a rate of 0.75–3 % and Fishoil at a rate of 1.5–6 % (Table 2) in the belief that these products could boost plant health and yield, and reduce the incidences of leaf diseases (based on a survey conducted by Samuelian 2014). Therefore, molasses in concentrations of 5, 10, and 20 %, Seasol in concentrations of 0.75, 1.5 and 3 %, and Fishoil in concentrations of 1.5, 3 and 6 % were tested as potential food sources for growth of *T. virens* and *T. harzianum* in vitro. Growth media consisted of the relevant concentrations of each food source in 15 % Bacto Agar (Becton, Dickinson and Company, Difco™). Furthermore, two plant derived adjuvants (wetting agents) were compared for their suitability to be used as spreader-stickers. These were NuFilm-17 and FleXstic at 2.4 μL/mL of PDA (Table 2). Measurements for all treatments were performed as described in 2.4.

### Survivability of *Trichoderma* spp. with a food substrate and a wetting agent under field conditions

Establishment and persistence of *Trichoderma* spp. populations on banana leaves was studied under field conditions at the CWTA (mean temperature 23.7 °C, 3334.6 mm year^−1^ rainfall, 136 days of rain ≥1 mm; soil: reddish brown light clay (Heiner and Smith 1987); 17.3 m elevation). The trial was conducted on bananas Musa (AAA, Cavendish subgroup) cv. ‘Williams’ irrigated by mini-sprinklers. The fertiliser program for the experiment consisted of biweekly applications of potassium nitrate (19.3 % *N*, 0 % *P* and 28.4 % *K*) at the rate of 35.7 kg/ha and biweekly applications of urea (15.7 kg/ha) through the mini-sprinkler irrigation system. No fungicides were applied during the experiment. *Trichoderma* isolates were cultured on PDA plates as already described under section ‘Fungal isolates and phylogenetic analysis’. Conidia collection and enumeration was conducted as described under section ‘Biological control of *Deightoniella torulosa* with *Trichoderma* spp. in vivo’ to a final concentration of 10^6^ spores/mL for each isolate. Treatments included *Trichoderma* spp., mixed in equal proportions, in water; *Trichoderma* spp. in 5 % molasses; and *Trichoderma* spp. in 5 % molasses and NuFilm-17 at 2.4 μL/mL. Each treatment was applied on two leaves of an individual plant on both the adaxial (upper) and abaxial (lower) surfaces of the leaves with a hand-held 500 mL spray bottle until runoff. Each treatment was replicated 4 times. Control consisted of leaves treated with water alone. Confirmation of the presence of *Trichoderma* populations was conducted by removing two 5–10 mm^2^ leaf sections from each leaf with a sterile scalpel blade every 48 h and culturing the leaf segments on PDA as described under section ‘Fungal isolates and phylogenetic analysis’. Evaluation of *Trichoderma* spp. was based on morphological characterisation where ‘0’ represented lack of observation of *Trichoderma* development and ‘1’ represented fungal growth. Presence of *Trichoderma* populations for each treatment was calculated as the sum of presence or absence of *Trichoderma* populations in the samples analysed divided by the number of samples (*n* = 8) and multiplied by 100 to represent the results as percentile revived *Trichoderma* populations. The experiment commenced at the end of March 2014 and was concluded at the end of April 2014. A second experiment was conducted in March–April 2015.

### Biological control of *Deightoniella torulosa* with *Trichoderma* spp. in vivo

A pilot study performed with the banana leaf pathogens analysed in this study determined that the most aggressive pathogen on leaves of young plants was *D. torulosa* causing the death of the infected plants within 2 weeks of infection (data not shown). Therefore, the potential of *Trichoderma* sp. to suppress *D. torulosa* using the protocol developed in this study was tested under glasshouse conditions. Tissue-cultured plants were potted on a potting mix (New Zealand peat: medium grade sand: coarse horticultural grade perlite 2:1:1; 2 g/L Growforce 101 fertiliser; 2 g/L Superphosphate; 4 g/L Dolomite) and grown in a humidity chamber at 26 °C for 14 days. Plants were further placed in a completely randomised design on a glasshouse bench and grown at ambient temperature 18–31 °C for 1 week. Plants were irrigated with overhead sprinklers. Infection of plants with conidia and fungal vegetative parts was conducted according to Alvindia (2012) with small modifications. One-month-old culture of *D. torulosa* grown on PDA was flooded with 20 mL sterile distilled water. Fungal colony was lightly scraped with a sterile Pasteur glass pipette to harvest fungal conidia and mycelium. The resulting suspension, containing 300 conidia/mL (determined as described under section “In vitro assessment of best formulations of food substrate and other adjuvants”), was transferred to a glass beaker and lightly mixed with a glass rod. The suspension was delivered to young banana plantlets with a pure bristle 38 mm paint brush until runoff as both the adaxial and abaxial surfaces of the leaves were treated. Banana plants were covered with black plastic bags for 48 h to stimulate pathogen infection. Treatments included *D. torulosa* alone, *D. torulosa* with 5 % molasses and NuFilm-17 at 2.4 μL/mL, and *D. torulosa* with co-application with *Trichoderma* spp. combined with 5 % molasses and NuFilm-17 at 2.4 μL/mL. Control plants were treated with water. Visible lesions were assessed 72 h post infection and a modified disease severity index (DSI; Stover and Dickson 1970) = [(Sum nb)/(*N* − 1) × *T*], was calculated, where *n* = number of leaves in each grade, *b* = grade, *N* = number of grades used (total of 4 as ‘0’ represents a leaf without symptoms, ‘1’—symptoms observed on 25 % of the leaf; ‘2’—symptoms observed on 50 % of the leaf; ‘3’—symptoms observed on 75 % of the leaf; and ‘4’—symptoms observed on 100 % of the leaf), and *T* = total number of leaves graded on each plant. Confirmation of infection was conducted by plating leaf areas with symptoms on PDA and the developed specie determined based on morphological and cultural characteristics. The experiment was conducted twice.

### Statistical analyses

ANOVA techniques were used to quantify statistical differences between treatments using Statistix 8 (Analytical Software: www.statistix.com). Tukey’s LSD all pairwise comparisons test was used for comparison of means at *P* = 0.05.

## Results

### Isolation and identification of *Trichoderma* spp.

**Fig. 1:**
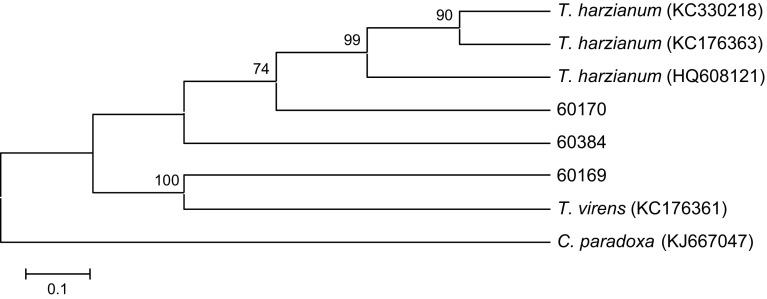
Phylogenetic relationship between *Trichoderma* isolates obtained during this study. Consensus tree was inferred using the neighbour-joining method. Sequences available at public databases were incorporated in the analyses. *Numbers* at nodes represent 1000 bootstrap replications. Only *values* above 70 % are indicated. The tree was rooted to *Ceratocystis paradoxa*. *Scale bar* represents genetic distances. NCBI accession numbers are presented in *brackets*

Based on phylogenetic/molecular analyses of the ITS1 conserved region of *Trichoderma* spp. (Fig. 1) the isolate BRIP60169 was identified as *Trichoderma* (*Hypocrea*) *virens*, and BRIP60170 and BRIP60384 as *Trichoderma* (*Hypocrea*) *harzianum* (Table 1).

### In vitro antagonism of *Trichoderma* spp. against banana leaf pathogens

Measured by the degree of coverage, BRIP60169 was the least antagonistic organism against *C. musae* and *D. torulosa* compared to BRIP60170 and BRIP60384 (Table 3). BRIP60170 and BRIP60384 displayed the same degree of coverage of all fungal pathogens studied. No difference between the antagonistic abilities of all *Trichoderma* isolates against *Mycosphaerella musicola* was observed.

**Table 3:** Coverage degrees of *Trichoderma* isolates against banana leaf pathogens using the confrontation technique in vitro on PDA plates incubated at 26 °C for 7 days

Fungal pathogen	*Trichoderma* isolate		
	BRIP60169	BRIP60170	BRIP60384
*Cordana musae*	3	2	2
*Deightoniella torulosa*	2	1	1
*Mycosphaerella musicola*	1	1	1
*Nigrospora sp.*	1.5	1	1

‘1’—*Trichoderma* completely overgrows the pathogen and covers the entire medium surface; ‘2’—*Trichoderma* overgrows two-thirds of the pathogen; ‘3’—*Trichoderma* overgrows half of the pathogen

### Assessment of fungicide sensitivity

**Fig. 2:**
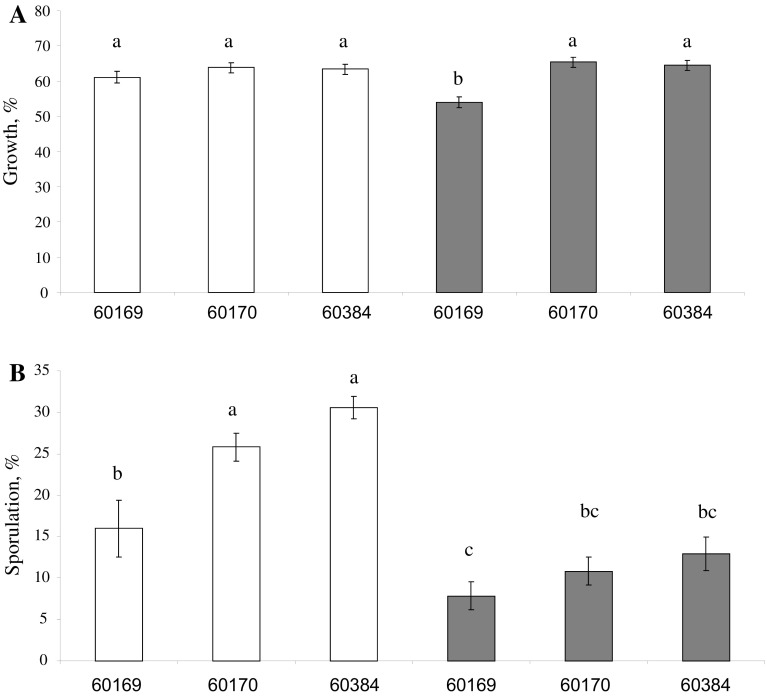
In vitro vegetative (*a*) and sporulation (*b*) sensitivity of *Trichoderma virens* (BRIP60169), and *T. harzianum* (BRIP60170 and BRIP60384) to Biopest oil at 2.5 L/ha (*white square*) and 5 L/ha (*grey square*) (Table 2). Results are presented in relation to fungal development on potato dextrose agar. *Error bars* indicate standard error. *Bars* with a *different letter* are significantly different according to Tukey’s LSD all-pairwise comparisons test at a 5 % similarity confidence level

Mancozeb and Mancozeb combined with Biopest Oil^®^ at 2.5 and 5 L/ha completely inhibited mycelium growth of all *Trichoderma* isolates analysed. Biopest Oil^®^ at 2.5 L/ha suppressed vegetative growth of the *Trichoderma* isolates by approximately 35 % (Fig. 2a). The same was observed for Biopest Oil^®^ at 5 L/ha for BRIP60170 and BRIP60384 but the growth rate of BRIP60169 was slower by 46 % compared to the isolate’s development on PDA. Biopest Oil^®^ at 2.5 L/ha reduced the number of spores of BRIP60170 and BRIP60384 by 69 and 74 %, respectively, when compared to their sporulation on PDA and of BRIP60169 by 84 % (Fig. 2b). Sporulation for all isolates on Biopest Oil^®^ at 5 L/ha was lower than for 2.5 L/ha. BRIP60169 produced the smallest number of spores on Biopest Oil^®^ at 5 L/ha.

### Food substrate and a spreader-sticker formulation

**Fig. 3:**
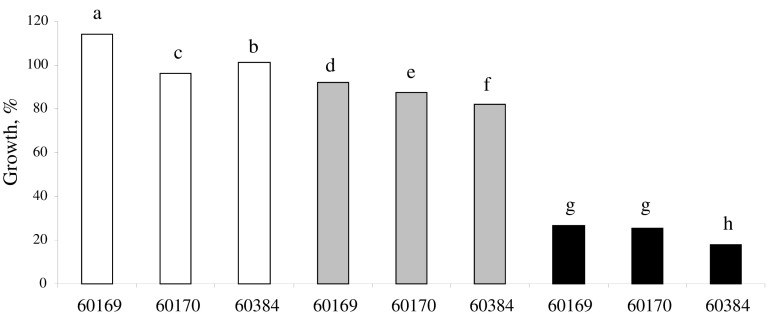
In vitro sensitivity of *Trichoderma virens* (BRIP60169), and *T.* *harzianum* (BRIP60170 and BRIP60384) to 5 % (*white square*), 10 % (*grey square*), and 20 % (*black square*) Molasses. Results are presented in relation to fungal development on Potato Dextrose Agar. Standard error values were <1 and are not presented on the graph. *Bars* with a *different letter* are significantly different according to Tukey’s HSD all-pairwise comparisons test at a 5 % similarity confidence level

**Fig. 4:**
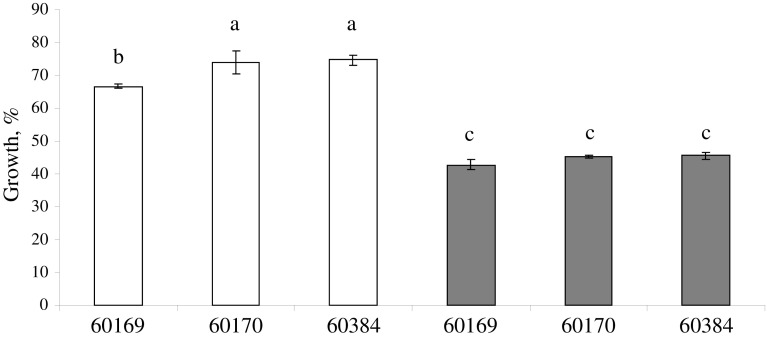
In vitro sensitivity of *Trichoderma virens* (BRIP60169), and *T. harzianum* (BRIP60170 and BRIP60384) to two non-ionic di-1-p-menthene additives—NuFilm-17 (*white square*) and FleXstic (*grey square*). Results are presented in relation to fungal development on potato dextrose agar. *Error bars* indicate standard error. *Bars* with a *different letter* are significantly different according to Tukey’s LSD all-pairwise comparisons test at a 5 % similarity confidence level

A very slow mycelium growth of the *Trichoderma* isolates analysed in this study was observed on 0.75 % Seasol (data not shown) but not on the higher concentrations tested. Growth was not observed on any of the concentrations of Fishoil. Mycelium growth for all *Trichoderma* isolates was equal or even faster at 5 % molasses compared to fungal development on PDA (Fig. 3). 10 and 20 % molasses was less favourable food source for the *Trichoderma* isolates compared to 5 % molasses. It was found that NuFilm-17 was less inhibitory for all *Trichoderma* spp. compared to FleXstic (Fig. 4).

### Survivability of *Trichoderma* spp. under field conditions

**Fig. 5:**
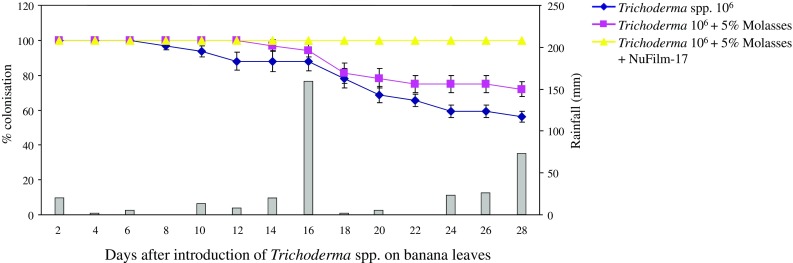
Survivability of *Trichoderma* spp. on banana leaves under natural conditions with a food substrate and a spreader-sticker. *Bars* represent rainfall in 48 h intervals in relation to the commencement of the experiment. *Error bars* represent standard deviations

Based on the fungicide sensitivity, antagonistic properties and growth rate of the three *Trichoderma* spp., and development on a food substrate and a spreader-sticker, it was decided to study the potential of BRIP60170 and BRIP60384 in combination with 5 % molasses and NuFilm-17 at 2.4 μL/mL to colonise banana leaves under natural conditions. *Trichoderma* spp. applied with 5 % molasses and NuFilm-17 were fully recovered during the course of the trial (Fig. 5). A decline in the *Trichoderma* spp. colonisation was observed after the sixth day of introduction of the organisms with water alone and after the twelfth day with 5 % molasses on banana plants under natural conditions. Subsequently *Trichoderma* spp. colonisation deteriorated for both treatments and dropped down to 56 % and 72 % for the *Trichoderma* spp. with water and *Trichoderma* spp. with 5 % molasses, respectively, after 28 days of application. During that period the total amount of rainfall received at the experimental site was 361.3 mm (http://www.bom.gov.au) with the highest amount of 160.2 mm received on the sixteenth day after the initiation of the experiment.

### Biological control of banana leaf diseases in vivo

The ability of *Trichoderma* spp. to suppress the banana fungal leaf pathogen *D. torulosa* was tested under greenhouse conditions. It was observed that the two *Trichoderma* isolates BRIP60170 and BRIP60384 combined with 5 % molasses and NuFilm-17 significantly inhibited the development of *D. torulosa* (Table 4) to levels similar to the untreated control.

**Table 4:** Effect of *Trichoderma* spp. on *Deightoniella torulosa* development following assessment of disease severity index 72 h after infection

Treatment	Disease Severity Index
*D. torulosa*	35.6 a
*D. torulosa* with 5 % molasses and NuFilm-17	28.9 a
Co-application with *Trichoderma* spp. with 5 % molasses and NuFilm-17	4.4 b
Control, no fungal infection	0 b

Different letters indicate significantly different values according to Tukey’s LSD all-pairwise comparisons test at a 5 % similarity confidence level

## Discussion

Demand for sustainability in food production is becoming a mega trend amongst consumers. Customers expect safer and ‘sustainable food’, which is acknowledged through tougher regulations and registrations of synthetic products. Fast urbanisation of areas in close vicinity to agricultural plantations limits the use of aerial chemical spraying thus constraining their use and increasing the price of application. Heavy reliance on small molecule systemic chemicals and their overuse can give rise to rapid build-up of resistance leading to loss of effective control as it has happened in many horticultural industries. Currently, there is an increased interest in the reduction of chemical residues and prevention of resistance development through utilisation of biological products particularly *Trichoderma* spp. which are believed to present the highest potential as a commercial biofungicides around the world (Harman 2006; Harman et al. 2004; Lorito et al. 2010; Mukherjee et al. 2014).

Most of the research conducted has investigated the potential of biological agents alone and little attention has been given to the development of integrated approaches as alternatives for systemic chemicals for the control of banana leaf pathogens. The advantages of such a strategy will translate into reduced disease pressure, fewer fungicide applications, reduced risks of resistance to fungicides in the pathogen population and enhanced environmental benefits. However, use of biological control of banana leaf diseases is challenging because of the polycyclic development of the crop, unfurling of young leaves every 6–12 days, and the presence of plants of different ages in the plantations. In addition, biological agents are influenced by the environment and have to compete with other organisms to survive. Therefore, in this study we investigated the potential of *Trichoderma* spp. as a part of a holistic approach to control the growth of banana leaf fungal pathogens in combination with non-chemical products such as a food source and a spreader-sticker and a ‘softer’ chemical product.

This study collated data needed for further advances in developing an integrated approach for a sustainable and environmentally friendly innovations in banana leaf disease protection. Antagonism of *Trichoderma* spp. against banana leaf pathogens, fungicide sensitivity, food formulation and adjuvant, and survivability under natural conditions were investigated. The results show that both *T.* *harzianum* and *T. virens* were effective in controlling banana leaf pathogens in vitro. The striking feature of *T. harzianum* is its rapid growth which enabled it to overgrow *Deightoniella torulosa*, *Cordana musae* and *Mycosphaerella musicola* within a week of dual incubation with a pathogen. Similar strong antagonistic abilities were reported for *T. harzianum* in controlling banana fungal pathogens (Alvindia 2013; Alvindia and Hirooka 2011; Alvindia and Natsuaki 2008).

Success of an integrated control depends on the survivability and compatibility of biological agents with all the products used in a management program which could be accomplished by alternation with a softer chemical. A level of control similar to that obtained with standard fungicides was achieved for *Botrytis cinerea* control when *T. harzianum* was alternated with dicarboximide fungicides (Elad et al. 1993a, b). Petroleum-derived mineral spray oil programs have been used by the banana industry worldwide since the 1950s. Oil retards initial fungal infection and development and enhances fungicide performance (Beattie et al. 2002). There has been a concern that accumulation of oil might cause phytotoxicity, which is enhanced by environmental conditions, especially at temperatures over 32 °C and high humidity. However, phytotoxicity has been associated with older mineral formulations while the possibility of photodegradation has been strongly reduced with the current highly refined oils. Biopest oil^®^ has been available for many years for Sigatoka control and is currently registered in Australia and routinely used along with Mancozeb for the control of yellow Sigatoka. In this study Biopest oil^®^ suppressed *Trichoderma* spp. in vitro, but did not completely inhibit their growth as Mancozeb. Therefore, a combination of biocontrol preparation with Biopest oil^®^ might be a feasible alternative towards reducing levels of fungicide residues and adding advantage for commercial application.

Survival of *Trichoderma* populations on the phylloplane is a key factor for achieving effective control (Elad et al. 1993a, b). McKenzie et al. (1991) speculated that poor effectiveness to suppress grey mould under field conditions could be explained by poor survival of the biological agent on the phylloplane. Food sources are used for mass production of *Trichoderma* spp. which afterwards are delivered as dried formulations (Thangavelu et al. 2004; Mukherjee et al. 2014). In the present study, an innovative approach to enhance the survivability and persistence of *Trichoderma* populations in the field through the addition of a food source and an adjuvant was investigated. NuFilm-17 is a surfactant spreader-sticker that was also reported to provide ultraviolet protection of entomopathogenic fungus *Beauveria bassiana* against the boll weevil, *Anthonomus grandis grandis* (Wright and Chandler 1992). NuFilm-17 is recommended for use with Sentinel, a *Trichoderma* spp. based biological control product supplied by Agrimm (http://www.agrimm.co.nz/) with the potential to protect grapevines against *Botrytis cinerea*. Arthurs and Lacey (2004) used NuFilm-17 spreader-sticker in all treatments during a field evaluation of commercial formulations of the coding moth granulovirus against natural infestations of codling moth in Pacific Northwest apple orchards even though the authors did not elaborate on the reasons behind this decision. Elad et al. (1993b) reported a delay in *Botrytis cinerea* development on ruscus when high rates of NuFilm-17 were applied. In this study a slight decline in *Trichoderma* spp. mycelium growth was observed when fungi were grown on di-1-p-menthene in vitro, which did not seem to have a negative effect on the *Trichoderma* spp. colonisation under natural conditions. The adjuvant also prevented a decline in the *Trichoderma* spp. populations compared to what happened with *Trichoderma* spp. alone and *Trichoderma* spp. with a food source, especially after the high amount of rainfall on the sixteenth day after the biological agents were introduced in the field.

Commercial success of *Trichoderma* biofungicides would depend on identification of native strains adapted to local conditions. Furthermore, development of an integrated approach that incorporates such *Trichoderma* spp. with a soft chemical product may present a feasible option for the control of leaf diseases on banana as well as on other horticultural crops. This study has demonstrated the advantage of adding a food substrate and an adjuvant to *Trichoderma* spp. to effectively colonise banana phyllosphere for a prolonged period of time. Furthermore, it was shown that *Trichoderma* spp. are effective in controlling banana fungal pathogens under controlled conditions. The next logical step should be to elucidate the potential of this system under commercial conditions.

## References

[CR1] Abadie C, Zapater M-F, Carlier J, Pignoleti L, Mourichon X (2008). Artificial inoculation on plants and banana leaf pieces with *Mycosphaerella* spp., responsible for Sigatoka leaf spot diseases. Fruit.

[CR2] Alvindia DG (2012). Inhibitory influence of biological agents, plant oils and an inorganic salt on *Mycosphaerella fijiensis* and *Cordana musae*, the casual pathogen of black Sigatoka and leaf spot of banana. Afr J Microbiol Res.

[CR3] Alvindia DG (2013). Sodium bicarbonate enhances efficacy of *Trichoderma harzianum* DGA01 in controlling crown rot of banana. J Gen Plant Pathol.

[CR4] Alvindia DG, Hirooka Y (2011). Identification of *Clonostachys* and *Trichoderma* spp. from banana fruit surfaces by cultural, morphological and molecular methods. J Mycol.

[CR5] Alvindia DG, Natsuaki KT (2008). Evaluation of fungal epiphytes isolated from banana fruit surfaces for biocontrol of banana crown rot disease. Crop Prot.

[CR6] Arthurs SP, Lacey LA (2004). Field evaluation of commercial formulations of the codling moth granulovirus: persistence of activity and success of seasonal applications against natural infestations of codling moth in Pacific Northwest apple orchards. Biol Control.

[CR7] Barnet HL, Hunter BB (1998) Illustrated genera of imperfect fungi, 4th edn. APS Press, The American Phytopathological Society, St. Paul, Minnesota

[CR8] Beattie A, Watson D, Stevens M (2002) Spray oils beyond 2000. In: Proceedings of a conference held from 25 to 29 October 1999 in Sydney, New South Wales, Australia, pp 1–627

[CR9] Bell DK, Wells HD, Markham CR (1982). *In vitro* antagonism of *Trichoderma* species against six fungal plant pathogens. Phytopathology.

[CR10] Benítez T, Rincón AM, Limón CM, Codón AC (2004). Biocontrol mechanisms of *Trichoderma* strains. Int Microbiol.

[CR11] Chaverri P, Samuels GJ (2001). *Hypocrea virens* sp. nov., the teleomorph of *Trichoderma virens*. Mycologia.

[CR12] Chin KM, Wirz M, Laird D (2001). Sensitivity of *Mycosphaerella fijiensis* from banana to trifloxystrobin. Plant Dis.

[CR13] Eckert JW, Sievert JR, Patnayake M (1994). Reduction of imazalil effectiveness against citrus green mold in California packinghouses by resistant biotypes of *Penicillium digitatum*. Plant Dis.

[CR14] Elad Y, Kirshner B, Gokkes M, Peer R (1993). Disease symptoms caused by Botrytis cinerea in Ruscus hypoglossum plants and their control. Phytoparasitica.

[CR15] Elad Y, Zimand G, Zaqs Y, Zuriel S, Chet I (1993). Use of *Trichoderma harzianum* in combination or alternation with fungicides to control cucumber grey mould (*Botrytis cinerea*) under commercial greenhouse conditions. Plant Pathol.

[CR16] Elad Y, Gullino ML, Shtienberg D, Aloi C (1995). Managing *Botrytis cinerea* on tomatoes in greenhouses in the Mediterranean. Crop Prot.

[CR17] González R, Bustamante E, Shannon P, Okumoto S, Leandro G (1996). Selección de microorganismos quitinolíticos en el control de la Sigatoka negra (*Mycosphaerella fijiensis*) en banano. Manejo Integrado Plagas.

[CR18] Grice K, Peterson RA (2002) Banana fungicide resistance. Final report. Horticulture Australia, FR99038, pp 1–51

[CR19] Harman GE (2000). Myths and dogmas of biocontrol: changes in perceptions derived from research on *Trichoderma harzianum* T-22. Plant Dis.

[CR20] Harman GE (2006). Overview of mechanisms and uses of *Trichoderma* spp. Phytopathology.

[CR21] Harman GE, Latorre B, Agosin A, San Martin R, Riegel DG, Nielsen PA, Tronsmo A, Pearson RC (1996). Biological and integrated control of Botrytis bunch rot of grape using *Trichoderma* spp. Biol Control.

[CR22] Harman GE, Howell CR, Viterbo A, Chet I, Lorito M (2004). *Trichoderma* species – opportunistic, avirulent plant symbionts. Nat Rev.

[CR23] Heiner IJ, Smith CD (1987) South Johnstone research station soils. Land Research Branch, Queensland Department of Primary Industries. D.P.I. Ref. No 86-112-P2596

[CR24] Holmes GJ, Eckert JW (1999). Sensitivity of *Penicillium digitatum* and *P. italicum* to postharvest citrus fungicides in California. Phytopathology.

[CR25] Jacobsen BJ, Zidack NK, Larson BJ (2004). The role of bacillus-based biological control agents in integrated pest management systems: plant diseases. Phytopathology.

[CR26] Jiménez JM, Galindo JJ, Ramírez C, Galindo JJ, Jaramillo R (1987). Estudios sobre combate biológico de Mycosphaerella fijiensis var. difformis mediante bacterias epífitas. Proceedings of ACORBAT meeting, 7th Centro Agronómico Tropical de Investigación y Enseñanza (CATIE).

[CR27] Karaoglanidis GS, Thanassoulopoulos CC, Ioannidis PM (2001). Fitness of *Cercospora beticola* field isolates—resistant and—sensitive to demethylation inhibitor fungicides. Eur J Plant Pathol.

[CR28] Lorito M, Woo SL, Harman GE, Monte E (2010). Translational research on *Trichoderma*: from omics to the field. Annu Rev Phytopathol.

[CR29] Marín DH, Romero RA, Guzmán M, Sutton TB (2003). Black Sigatoka: an increasing threat to banana cultivation. Plant Dis.

[CR30] McKenzie LL, Benzi D, Dellavalle D, Gullino ML (1991). Survival on the phylloplane of strains of *Trichoderma* spp. antagonistic to *Botrytis cinerea*. Petria.

[CR31] Miranda JE (1996) Evaluación de microorganismos antagonistas al hongo *Mycosphaerella fijiensis* Morelet, colocados en el interior y exterior de la planta de banano. Mag. Sci. thesis. CATIE, Turrialba, Costa Rica

[CR32] Mondal SN, Bhatia A, Turksen S, Timmer LW (2005). Baseline sensitivities of fungal pathogens of fruit and foliage of citrus to azoxystrobin, pyraclostrobin, and fenbuconazole. Plant Dis.

[CR33] Monte E (2001). Understanding Trichoderma: between biotechnology and microbial ecology. Int Microbiol.

[CR34] Mukherjee AK, Sampath Kumar A, Kranthi S, Mukherjee PK (2014). Biocontrol potential of three novel *Trichoderma* strains: isolation, evaluation and formulation. 3 Biotech.

[CR35] Pitt WM, Huang R, Steel CC, Savocchia S (2010). Identification, distribution and current taxonomy of Botryosphaeriaceae species associated with grapevine decline in New South Wales and South Australia. Aust J Grape Wine Res.

[CR36] Romero RA, Sutton TB (1996). Sensitivity of *Mycosphaerella fijiensis*, causal agent of black Sigatoka of banana, to propiconazole. Phytopathology.

[CR37] Romero RA, Sutton TB (1998). Characterization of benomyl resistance in *Mycosphaerella fijiensis*, cause of black Sigatoka of banana, in Costa Rica. Plant Dis.

[CR38] Rossman AY, Seifert KA, Samuels GJ, Minnis AM, Schroers H-J, Lombard L, Crous PW, Põldmaa K, Cannon PF, Summerbell RC, Geiser DM, Zhuang W-Y, Hirooka Y, Herrera C, Salgado-Salazar C, Chaverri P (2013). Genera in *Bionectriaceae*, *Hypocreaceae*, and *Nectriaceae* (*Hypocreales*) proposed for acceptance or rejection. IMA Fungus.

[CR39] Samuelian S (2014). Grower survey—how we manage yellow Sigatoka? *Australian Bananas*, Issue 41. Autumn Winter.

[CR40] Samuelian S, Greer LA, Cowan K, Priest M, Sutton TB, Savocchia S, Steel CC (2012). Phylogenetic relationships, pathogenicity and fungicide sensitivity of *Greeneria uvicola* isolates from *Vitis vinifera* and *Muscadinia rotundifolia* grapevines. Plant Pathol.

[CR41] Samuelian S, Greer LG, Savocchia S, Steel CC (2014) Application of Cabrio (a.i. pyraclostrobin) at flowering and véraison reduces the severity of bitter rot (*Greeneria uvicola*) and ripe rot (*Colletotrichum acutatum*) of grapes (*Vitis vinifera*). Aust J Grape Wine Res doi:10.1111/ajgw.12073

[CR42] Schilly A, Chaves N, Guzmán M, Sandoval J, Staver C, Dita M (2014) Exploring root-associated entophyte microorganisms from *Musa* spp. for enhancing plant health. 29th International horticultural congress, 17–22 August 2014, Brisbane, Australia

[CR43] Sholberg PL, Haag PD (1993). Sensitivity of Venturia inaequalis isolates from British Columbia to flusilazole and mycobutanil. Can J Plant Pathol.

[CR44] Sierotzki H, Parisi S, Steinfeld U, Tenzer I, Poirey S, Gisi U (2000). Mode of resistance to respiration inhibitors at the cytochrome bc_1_ enzyme complex of *Mycosphaerella fijiensis* field isolates. Pest Manag Sci.

[CR45] Stover RH (1977). Behavior of benomyl tolerant strains of the black Sigatoka pathogen in the field. Proc Am Phytopathol Soc.

[CR46] Stover RH, Dickson JD (1970). Leaf spot of banana caused by *Mycosphaerella musicola*: methods of measuring spotting prevalence and severity. Trop Agric (Trinidad).

[CR47] Stover RH, Slabaugh WR, Grove MD (1978). Effect of chlorothalonil on a severe outbreak of banana leaf spot caused by benomyl tolerant *Mycosphaerella fijiensis* var. difformis. Phytopathol News.

[CR48] Tamura K, Dudley J, Nei M, Kumar S (2007). MEGA4: molecular evolutionary genetics analysis (MEGA) software version 4.0. Mol Biol Evol.

[CR49] Thangavelu R, Palaniswami A, Velazhahan R (2004). Mass production of *Trichoderma harzianum* for managing fusarium wilt of banana. Agric Ecosyst Environ.

[CR50] White TJ, Bruns TD, Lee S, Taylor JW, Innis MA, Gelfand DH, Sninsky JJ, White TJ (1990). Amplification and direct sequencing of fungal ribosomal RNA genes for phylogenetics. PCR protocols: a guide to methods and applications.

[CR51] Wright JE, Chandler LD (1992). Development of a biorational myocoinsecticide—Beauveria-bassiana conidia formulation and its application against boll-weevil populations (Coleoptera, Curculionidae). J Econ Entomol.

